# The predictive value of eGFR combined with BNP detection in acute kidney injury after acute myocardial infarction

**DOI:** 10.4314/ahs.v23i2.62

**Published:** 2023-06

**Authors:** Chen-Yu Geng, Fang-Ze Wang, Rui Zhang, Yan-Yu Liu, Jian Wang

**Affiliations:** 1 School of Clinical Medicine, Weifang Medical University, Weifang 261000, China; 2 Department of Cardiology, Weifang People's Hospital, Weifang 261000, China

**Keywords:** AMI, AKI, eGFR, BNP, predictive value

## Abstract

**Objective:**

The combined detection of eGFR and BNP may provide some value in predicting the occurrence of AKI after AMI, and the study is designed to propose and validate this hypothesis.

**Methods:**

In retrospective research, AMI patients hospitalized at Weifang People's Hospital from January to December 2020 were included. Whether AKI occurred within a week of admission, patients were divided into two groups. Clinical data from two groups of patients were collected, and the Logistic regression model analysed the risk factors for AKI after AMI. The association between eGFR and BNP was analysed using Pearson linear correlation. The predictive value of eGFR and BNP alone and combined detection on AKI after AMI was analysed using the receiver operating characteristic (ROC) curve.

**Results:**

Multivariate logistic regression showed that eGFR, BNP, HDLC, UA, and K ions were AKI risk factors (P < 0.05). The eGFR correlates negatively with BNP (R = -0.324, P < 0.05). The area under the curve (AUC) of eGFR and BNP alone and combined prediction for post-AMI AKI were 0.793, 0.826, and 0.831, respectively.

**Conclusion:**

The combined detection of eGFR and BNP has a high predictive value for AKI development in AMI patients.

## Introduction

Acute myocardial infarction (AMI) is the necrosis of the heart muscle resulting from acute and chronic ischemia and hypoxia of the coronary artery. It is a common and life-threatening severe syndrome in clinical practice. The prevalence of AMI has risen in the last 20 years. Acute kidney injury (AKI) is a condition with clinical manifestations that occurs when renal function declines rapidly over a short period of time due to various causes. Studies have shown that the frequency of AKI among hospitalized AMI patients ranged from 7.1% to 29.3%^1^. In addition, when AMI is worsened by cardiogenic shock, the incidence of AKI exceeds fifty percent^2^. Among patients with AMI, the mortality rate in people with AKI is 20 to 40 times greater than in individuals without AKI. AKI patients also experienced higher long-term sequelae, such as recurrent AMI, heart failure, chronic renal disease development, and long-term death^3^. How to early identify and prevent post-AMI AKI has become a critical clinical research topic, which has far-reaching significance for improving prognosis. Current studies indicate that the causes of AKI after AMI are very complex and may involve many mechanisms, such as ischemia-reperfusion injury, inflammation, oxidative stress, nephrotoxicity, etc^4^; the prediction of post-AMI AKI is being widely carried out, such as the combined detection of red blood cell distribution width (RDW) and N-terminal brain natriuretic peptide (NT-proBNP)^5^. There are no straightforward, useful clinical markers for post-AMI AKI. eGFR and BNP are easily detectable, hence the author used them to predict AKI after AMI. eGFR measures the kidney's ability to eliminate metabolic wastes and toxins. Acute renal damage reduces eGFR. BNP is a natural hormone produced by cardiomyocytes and expressed in ventricular muscle and brain tissue. Due to ventricular dilatation, it's rapidly created and released into the circulation when the left ventricle malfunctions. It regulates cardiac function and is removed by glomerular filtration. AMI patients are often accompanied by crdiac insufficiency, BNP is often several times higher than the average value, and BNP increases significantly when AKI is accompanied^6^. It is reasonable to speculate a specific correlation between BNP and AKI. Given their different clearance mechanisms in the body, this research focuses on the bioactive hormone BNP, not NT-proBNP. This study aims to establish the predictive utility of eGFR and BNP for AKI following AMI and propose novel diagnostic and therapy suggestions.

## Methods

### General Information

A retrospective study was conducted on 200 AMI patients admitted to Weifang People's Hospital from January 2020 to December 2020.

(1) **The diagnostic criteria for AMI** 7: (1)Typical chest pain, duration ≥ 30 min; (2) Accompanied by obvious myocardial ischemia; (3) Electrocardiogram showed the presence of new ischemic lesions; (4) With pathological Q wave; (5) The activity of cardiomyocytes was obviously lost, and the local wall activity was abnormal; (6) Coronary artery thrombosis.

(2) **Inclusion criteria:** (1) Meet the diagnostic criteria of AMI, age ≥18; (2) No loss of clinical data; (3) All patients underwent renal function examination; (4) the state of consciousness is clear; (5) Voluntarily sign informed consent; (6) Normal cognitive function, good communication skills.

(3) **Exclusion conditions:** (1) Insufficient initial clinical data; (2) Death or discharge due to insufficient AKI development time within 48 hours after admission; (3) Complications of septic shock; (4) the previous diagnosis was end-stage renal disease. One hundred forty-one males and 59 females aged 21-90 years, with an average of (61.07 ±12.19) years. The number of lesions: One branch 24 cases, two branches 59 cases, three branches 117 cases. (4) Diagnosis and grouping criteria for post-AMI AKI: based on KDIGO criteria^1^,

**AKI diagnosis was defined as:** (10 SCr increase > 0.3mg/dL within 48 hours; (2) Increase ≥ 1.5 times from baseline in the first seven days. AKI can be diagnosed if any of the above criteria are met. AMI patients were classified into the AKI group (n = 50) and the non-AKI group (n = 150) based on whether AKI developed within a week of admission. The clinical indicators of the AKI group and non-AKI group were compared to analyse the risk factors of AKI after AMI and observe the correlation of eGFR and BNP and the value of their combination in predicting the occurrence of AKI after AMI.

### Clinical data collection

Clinical data were collected, including gender, the number of lesions, age, smoking history, history of diabetes, hypertension, history of alcohol consumption, use of diuretics, brain natriuretic peptide (BNP), estimated glomerular filtration rate (eGFR), high-density lipoprotein cholesterol (HDLC), uric acid (UA), D Dimer, low-density lipoprotein cholesterol (LDLC), Prothrombin Time (PT), triglyceride (TG), total cholesterol (TC), serum creatinine (SCr), red blood cell distribution width (RDW), blood urea nitrogen (BUN), sodium ion (Na+), potassium ion (K+), left atrium diameter (LAD), left ventricular ejection fraction (LVEF), interventricular septum thickness (IVST), and left ventricular end-diastolic diameter (LVEDD).

### Statistical Analysis

The program SPSS 26.0 was used to analyse the data. A t-test was performed on the measurement data, which was represented as X ± S. The rate of counting data (%) was expressed as a chi-square test. The risk variables for AKI following AMI were studied using a multivariate logistic regression model. Pearson linear correlation analysed the correlation between eGFR and BNP in patients with AMI and AKI. The area under the curve (AUC) was calculated after the receiver operating characteristic curve (ROC) was created, and the value of eGFR and BNP detection alone or jointly in predicting the occurrence of AKI after AMI was analysed. P < 0.05 was considered significance in the statistical sense.

## Results

### Comparison of clinical data, eGFR, and BNP between the two groups

The results indicated that BNP levels in the AKI group (362.36 ± 13.06 pg/mL) were considerably greater than in the non-AKI group (207.95 ± 13.60 pg/mL). There was a statistically significant difference (P < 0.01). The eGFR was considerably lower in the AKI group [81.09 ± 3.54 mL/ min·1.73 m^2^)] than in the non-AKI group [99.12 ± 1.67 mL/ min-1.73 m^2^)], and there was a statistically significant difference (P < 0.01); The levels of other indicators such as UA, SCr and K+ were significantly higher in the AKI group compared to the non-AKI group. AKI patients also have lower HDLC and Na+ levels than the non-AKI patients (P < 0.05). The proportion of hypertension in the AKI group was 70%, which was significantly higher than that in the non-AKI group (42.67%) (P < 0.05; Table 1); the difference was statistically significant.

**Table 1:** Clinical data, eGFR and BNP were compared between the two groups

indicators	AKI group (n = 50)	The AKI group (n = 150)	T/chi square	P
Male / [cases (%)]	31 (62.00%).	110 (73.33%)	2.316	0.128
Female / [cases (%)]	19 (38.00%).	40 (26.67%).		
Age /	59.38 ± 11.49	61.63 ± 12.40	1.133	0.259
Number of lesions/cases (%)	—	—	1.056	0.590
one	4 (8.00%)	20 (13.33%).	—	—
two	16 (32.00%).	43 (28.67%).	—	—
three	30 (60.00%)	87 (58.00%)	—	—
History of hypertension / [cases (%)]	35 (70.00%).	64 (42.67%)	11.208	0.001
Diabetes history / [cases (%)]	13 (26.00%).	34 (22.67%).	0.232	0.630
Smoking history / [cases (%)]	26 (52.00%).	84 (56.00%)	0.242	0.622
History of alcohol consumption / [cases (%)]	23 (46.00%).	50 (33.33%).	2.596	0.107
Diuretics used / [cases (%)]	21 (42.00%).	47 (31.33%).	1.901	0.168
HDLC/(mmol/L)	1.02 ± 0.04	1.10 ± 0.02	2.011	0.046
LDLC / (mmol/L)	2.71 ± 0.12	2.89 ± 0.08	1.221	0.224
TC/(mmol/L)	4.44 ± 0.15	4.65 ± 0.09	1.173	0.242
TG/(mmol/L)	1.16 ± 0.17	1.22 ±1.10	0.292	0.771
UA/(µmol/L)	415.78 ± 12.94	333.76 ± 7.72	5.355	0.000
BUN/(mmol/L)	6.10 ± 0.58	6.29 ± 0.24	0.342	0.733
SCr/ ([µmol/L)	75.28 ± 2.33	63.81 ± 2.43	2.592	0.010
eGFR/[mL/(min-1.73 m^2^)]	81.09 ± 3.54	99.12 ± 1.67	5.095	0.000
RDW/%	41.19 ± 0.48	41.61 ± 0.25	0.801	0.424
BNP (pg/mL)	362.36 ± 13.06	207.95 ± 13.60	8.19	0.000
D-dimer (ng/L)	2499.92 ± 291.44	1688.91 ± 516.28	0.89	0.375
PT (s)	13.63 ± 0.37	13.31 ± 0.10	1.193	0.235
Na^+^(mmol/L)	137.92 ± 0.64	139.31 ± 0.31	2.123	0.035
K+(mmol/L)	4.29 ± 0.07	4.04 ± 0.03	3.601	0.000
IVST/mm	9.57 ± 0.15	9.38 ± 0.09	1.062	0.289
LVEDD/mm	50.66 ± 0.90	50.06 ± 0.41	0.677	0.499
LAD/mm	33.16 ± 0.58	33.31 ± 0.35	0.207	0.836
LVEF	57.28 ± 1.20	57.61 ± 0.67	0.242	0.809

### Risk factors analysis of AKI in patients with AMI

Logistic regression analysis, both univariate and multivariate of statistically significant variables in Table 1 showed that eGFR (P = 0.01), BNP (P = 0.000), HDLC (P = 0.036), UA (P = 0.002), K+ (P = 0.005) were AKI risk factors in AMI patients (P < 0.05; Table 2).

**Table 2:** Risk factors analysis of AKI in AMI patients

Variable	B	Standard error of	Wald	Degrees of freedom	Significant	Exp(B)
A history of high blood pressure	0.839	0.456	3.385	1	0.066	2.314
				
HDLC	1.968	0.938	4.403	1	0.036	0.140
	
UA	0.007	0.002	9.680	1	0.002	1.008
	
SCR	0.029	0.015	3.635	1	0.057	0.972
		
eGFR	0.040	0.016	6.623	1	0.010	0.961
	
BNP	0.005	0.001	12.585	1	0.000	1.005
				
Na+	0.086	0.053	2.643	1	0.104	0.918

K+	1.441	0.508	8.043	1	0.005	4.225

constant	7.913	8.157	0.941	1	0.332	2733.562

### Correlation analysis between eGFR and BNP in patients with AMI and AKI

**Figure 1:**
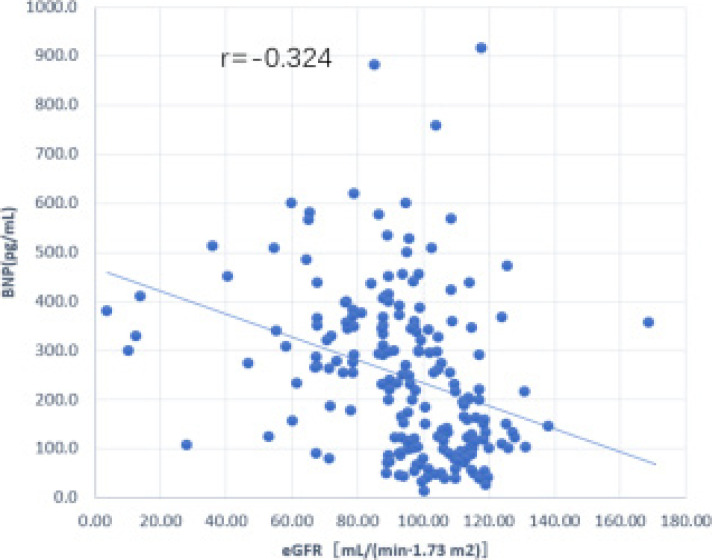
Linear correlation between eGFR and BNP in patients with AMI and AKI

In terms of risk factors for AKI in AMI patients, Pearson linear analysis showed that eGFR was negatively correlated with BNP in AMI patients with AKI (r = -0.324, P < 0.01; Figure 1).

### Predictive value analysis of eGFR and BNP on the occurrence of AKI after AMI

**Figure 2:**
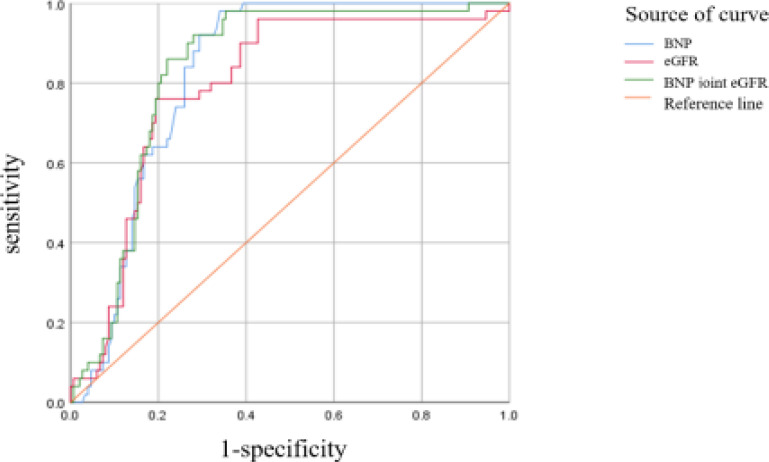
ROC curve of eGFR and BNP in predicting AKI after AMI

The AUC of eGFR and BNP detection alone and in combination for predicting the occurrence of AKI after AMI were 0.793, 0.826, and 0.831, respectively (Table 3). The ROC curve is shown in Figure 2. The predictive value of eGFR and BNP combined detection for AKI in AMI patients was superior to eGFR and BNP alone.

**Table 3:** A predictive value analysis of BNP and eGFR on the occurrence of AKI after AMI

indicators	AUC	Standard error	P	95% CI	Cut off value	The sensitivity / %	Specific degrees / %
BNP (pg/mL)	0.826	0.028	0.000	0.771 ~ 0.881	220	98.000	66.000
		
eGFR [mL/(min 1.73 m^2^)]	0.793	0.036	0.000	0.723 ~ 0.862	89.72	76.000	81.000
				
The BNP joint eGFR	0.831	0.030	0.000	0.772 ~ 0.891	—	92.000	72.000

## Discussions

AKI is a significant and possibly fatal complication of AMI (AMI). It has a high short-term and long-term death rate and a dismal prognosis, however it is potentially avoided. Post-AMI AKI is difficult and poorly understood. Early identification and prediction in clinical practice need simple, effective procedures.

Research shows, eGFR and BNP are risk factors for post-AMI AKI. Post-AMI AKI is connected to HDLC, UA, and K+ levels. Numerous studies show that UA increases when kidney injury occurs^8^. This study found that individuals with AKI following AMI had higher serum K+ levels than those without AKI. This may be due to poor K+ filtration through the glomeruli during AKI. HDLs prevent endothelial dysfunction and damage by lowering endothelial cell activation and adhesion molecule synthesis, activating endothelial nitric oxide synthase, and stimulating endothelium repair^9^, which may be beneficial to reverse the occurrence of AKI. Low HDL cholesterol may promote organ failure, especially postoperative AKI, in heart surgery patients^10^. This study indicated that AKI patients' serum HDLC concentration was lower than those without AKI, which was consistent with earlier research.

This study found that in AMI patients with AKI, eGFR was lower and BNP levels were greater. The two values were negatively associated, and the combined detection of the two had a significant predictive value for post-AMI AKI. Existing studies suggest that baseline eGFR reduction is a strong independent predictor of AKI^11^. In AMI patients, eGFR in individuals with AKI was lower than that in individuals without AKI. However, it was still in the lower limit of the normal range (81.09±3.54), which may indicate that eGFR, on average or slightly lower than the standard value, has a specific role in the incidence of AKI. Low eGFR has been linked to a higher risk of complications in people with coronary artery disease, according to research^12^. In this study, eGFR was reduced in patients with post-AMI AKI, which was in line with the findings of earlier research, which may be related to damage to the glomerular mechanical barrier and electrostatic barrier in patients with post-AMI AKI. Studies have shown that^13^ the predictive significance of natriuretic peptide levels in cardiac surgery is likely related to their ability to measure modest changes in left and right ventricle functions in systolic or diastolic problems, symptomatic or not. Ventricular wall stress induces natriuretic peptide secretion in volume expansion or pressure overload. Natriuretic peptide has predictive potential in disorders involving hemodynamic stress, except heart failure^14^. In this study, plasma BNP levels in post-AMI AKI patients were greater than in non-AKI patients, which may be related to hemodynamic stress caused by AKI.

This study also has limits: First of all, this study only collected significant patient information during hospitalization, not outside. Secondly, the sample size is modest; Finally, although this study suggests that a slight decrease in eGFR can predict AKI, it is based on previous detection data, and prospective experimental trials are lacking.
